# Pretraining improves prediction of genomic datasets across species

**DOI:** 10.1093/bioinformatics/btag139

**Published:** 2026-03-23

**Authors:** Fangrui Huang, Yitong Wang, Ashok Cutkosky, Janet H T Song

**Affiliations:** Department of Computer Science, Boston University, Boston, MA, 02215, USA; Department of Computer Science, Stanford University, Stanford, CA, 94305, USA; Department of Computer Science, Boston University, Boston, MA, 02215, USA; Department of Electrical and Computer Engineering, Boston University, Boston, MA, 02215, USA; Department of Human Evolutionary Biology, Harvard University, Cambridge, MA, 02138, USA

## Abstract

**Motivation:**

Recent studies suggest that deep neural network models trained on thousands of human genomic datasets can accurately predict genomic features, including gene expression and chromatin accessibility. However, training these models is computation- and time-intensive, and datasets of comparable size do not exist for most other organisms.

**Results:**

Here, we identify modifications to an existing state-of-the-art model that improve model accuracy while reducing training time and computational cost. Using this streamlined model architecture, we investigate the ability of models pretrained on human genomic datasets to transfer performance to a variety of different tasks. Models pretrained on human data but fine-tuned on genomic datasets from diverse tissues and species achieved significantly higher prediction accuracy while significantly reducing training time compared to models trained from scratch, with Pearson correlation coefficients between experimental results and predictions as high as 0.8. Further, we found that including excessive training tasks decreased model performance and that this decrease could be partially but not completely rescued by fine-tuning. Thus, simplifying model architecture, applying pretrained models, and carefully considering the number of training tasks may be effective and economical techniques for building new models across data types, tissues, and species.

**Availability and implementation:**

Code is available on GitHub and Figshare: https://github.com/optimizedlearning/genomicsML, https://doi.org/10.6084/m9.figshare.31796116.

## 1 Introduction

Determining how noncoding genomic sequences regulate the expression of nearby genes is critical to understanding how species, cell types, and disease states arise. Unfortunately, identifying the noncoding sequences that act as regulatory elements in a particular cell type or paradigm has proven to be incredibly challenging. A standard empirical approach to identify active noncoding sequences uses DNA accessibility and biochemical marks such as histone modifications as proxies for functional activity (Shlyueva *et al.* 2014). However, these experiments are expensive, may require samples that are not easily accessible, and cannot be feasibly performed on all the sequence differences that exist within normal and disease variation in the same species or that have arisen between species across evolutionary time. Thus, there has been great interest in harnessing existing genomic datasets to build machine learning models that can predict features like DNA accessibility and histone modifications directly from DNA sequence alone.

Recent efforts to predict genomic features directly from the underlying DNA sequence take inspiration from the dramatic successes of deep learning models in natural language processing and apply similar models to genomics settings (Alipanahi *et al.* 2015, Zhou and Troyanskaya 2015, Avsec *et al.* 2021a,b, Linder et al. 2023). One challenge in building models that predict genomic features from the underlying DNA sequence is handling long-range interactions between DNA segments that are up to 100s of kilobases (kb) away from each other. Classical neural network building blocks like convolutional layers are not well suited to long-range modeling, but the newer self-attention mechanism (Vaswani *et al.* 2017) directly addresses this limitation. Self-attention is the core component of modern language and computer vision models such as GPT (Radford *et al.* 2019), and is a natural candidate to improve genomic modeling by more effectively considering long-range interactions.

One recent model that employs self-attention to incorporate long-range sequence information is Enformer (Avsec *et al.* 2021a). This model combines both convolutional and self-attention blocks to predict 5313 human genomic datasets and 1643 mouse genomic datasets directly from 196 kb input DNA sequences. The Enformer model increases information flow by considering sequences that are up to 196 kb from the transcription start site, achieving an impressive 0.625 Pearson correlation coefficient between experimental results and predictions. However, the model has 246 million parameters and is extremely memory and compute intensive to train: the published version was trained on a TPU cluster for 192 TPU days with thousands of human and mouse datasets, and numerous additional resources were likely expended to iterate on different model and data choices. This computationally expensive process makes these techniques inaccessible to many researchers, who may not have the data, monetary resources, or hardware required to train such a model.

We wondered (i) whether it may be possible to reduce the size of the Enformer model such that it can fit into less advanced (and more commonly available) hardware without compromising model accuracy, (ii) whether we can utilize existing Enformer-like models to train new models in different paradigms (pretraining/fine-tuning), and (iii) what amount of training data is needed for high accuracy.

“Pretraining” refers to first training a model on a large amount of data and then using the weights from this initial training as the starting point for fine-tuning on a task of interest. This pretraining/fine-tuning process is already a standard technique in other areas of machine learning (i.e. computer vision and natural language processing), where it has been used to solve image recognition tasks, question answering tasks, and sentence completion tasks (Devlin *et al*. 2019, Dosovitskiy *et al*. 2021) using far less data and compute in the fine-tuning stage than would have been required to train a new model from scratch. This strategy has now begun to be applied to machine learning models that predict genomic features (Yuan *et al.* 2025), but systematic analyses of how best to perform pretraining and whether pretraining can be applied even for datasets that are very different from the initial training data have been limited. As a challenging, but biologically relevant paradigm, we considered whether pretraining on an Enformer-like model built from thousands of genomic datasets in one species (human) can improve accuracy when a new model is trained on only a single GPU using only one or a small number of genomic datasets in another species.

In this paper, we find that a simplified attention-based architecture derived from Enformer can be trained and fine-tuned on readily available academic hardware to high accuracy (Section 3.1). We then use our simplified architecture to examine models pretrained on human datasets and fine-tuned on datasets from disparate species. Strikingly, we find that this pretraining/fine-tuning process significantly increases accuracy across a wide variety of tasks, tissues, and species while reducing computation time compared to models trained from scratch (Sections 3.2 and 3.3). Finally, we find a tradeoff between generalization and specialization where training on an excessive number of tasks compromises model performance (Section 3.4). The strategies and techniques demonstrated here can help democratize the use of these models across researchers and experimental contexts and should be generalizable to other models, such as Borzoi (Linder *et al.* 2025) and Xpresso (Agarwal and Shendure 2020).

## 2 Materials and methods

We developed a modified version of the Enformer architecture that can be trained efficiently on a single 16GB Nvidia V100 GPU. We then pretrained this architecture on a large human genomic dataset from ENCODE for 10 epochs. This pretrained model was employed on a variety of downstream fine-tuning tasks involving smaller nonhuman genomic datasets. These fine-tuned models were compared to models trained from scratch on the smaller nonhuman genomic datasets.

### 2.1 Model architectures

**Figure 1:**
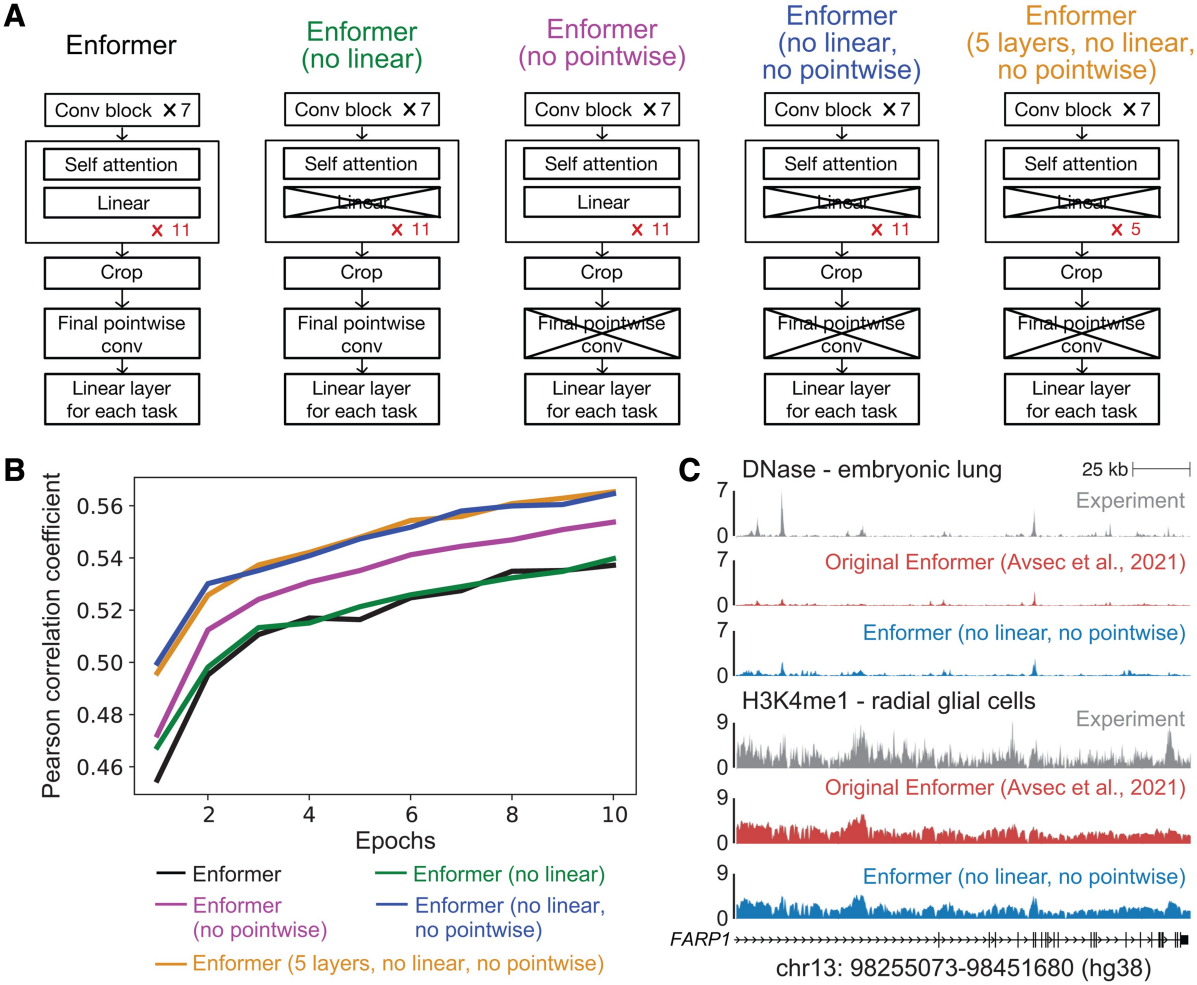
Comparison of different model architectures. (A) We compared model architectures for the pretraining model that had different numbers of attention blocks (11 or 5), were with or without the linear layer in the attention block, and were with or without the final pointwise convolutional layer. (B) Validation Pearson correlation coefficients for different model architectures. Models were trained for 10 epochs on all human datasets that were used to train Enformer. Maximum Pearson correlation coefficient: Enformer = 0.537, Enformer (no linear)=0.540, Enformer (no pointwise)=0.554, Enformer (no linear, no pointwise)=0.565, Enformer (5 layers, no linear, no pointwise)=0.565. (C) Representative examples of observed and predicted genomic tracks (normalized read depth).

The Enformer model consists of 7 convolutional residual blocks, 11 self-attention blocks, a pointwise convolutional layer and 2 linear “head” layers, corresponding to mouse and human tracks (Fig. 1A). The input to the model is a one-hot encoded DNA sequence of length 196,608 bp where each bp is represented as a 4-dimensional vector: A=[1,0,0,0], C=[0,1,0,0], G=[0,0,1,0], T=[0,0,0,1], N=[0,0,0,0]. For human input data, the output of the model provides predictions for 5313 tracks recorded at 128 bp resolution over the middle 114,688 bp of the input, corresponding to 114,688/128 = 896 values per track. For mouse input data, the model provides predictions for 1643 tracks. Thus, the output size for the human head is (896, 5313) and the output size for the mouse head is (896, 1643).

As a baseline, we trained the original Enformer model on only the human dataset for 10 epochs. We then trained several variants of the Enformer architecture also for 10 epochs on the human dataset. These variants were: (i) removing the final pointwise convolutional layer, (ii) removing a linear layer from the attention blocks, (iii) removing both the final pointwise convolutional layer as well as the linear layer from the attention blocks, and (iv) reducing the number of attention blocks from 11 to 5. Of these, we used variant (iii) for all downstream analyses.

### 2.2 Training data

Our pretraining data are subsets of the data developed by Kelley (2020) and modified by Avsec *et al.* (2021a) for training Enformer. This dataset has 34,021 training examples, each consisting of a length 196,608 DNA sequence from hg38, paired with 5313 label tracks at 128 bp resolution over the middle 114,688 bp of the input sequence.

As fine-tuning data, we employed genomic datasets collected from chickens, rhesus macaques, cows, pigs, and mice. These dataset are further divided into two types of assays: ATAC-seq data and H3K4me3 ChIP-seq data. ATAC-seq identifies regions of open chromatin commonly found at noncoding sequences that act as promoters or enhancers. H3K4me3 is a histone mark that is commonly found at promoters and enhancers (Shlyueva *et al.* 2014).

All data were preprocessed using the code developed by Kelley *et al.* (2018). Briefly, for each organism, we split chromosomes into shorter contiguous regions using the same data preprocessing pipeline used to generate the training data for Enformer. We then performed a second preprocessing step to identify regions that are homologous to regions in the original Enformer training data; we aligned sequences to the human genome using pslMap (Zhu *et al.* 2007) and assigned regions that had more overlap with sequences in the human training set than the human validation set to the training set and vice versa. This procedure generated validation and test sets with very low homology to the human training set (Fig. S1, available as supplementary data at *Bioinformatics* online).

The ATAC-seq data were collected in mice, cows, and pigs (Halstead *et al*. 2020, Kern *et al.* 2021). Training data were subsampled to 8936 sequences for each species to balance the training set sizes. Each sequence is paired with 11 ATAC-seq label tracks for mice, 13 for cows, and 13 for pigs. The Pearson correlation coefficient between replicate ATAC-seq datasets is 0.97 ± 0.044, placing an upper bound on the accuracy of these experiments.

The H3K4me3 ChIP-seq data were collected in mice, chickens, and rhesus macaques (Vermunt *et al*. 2016, Kelley *et al.* 2018, Halstead *et al*. 2020). The mouse H3K4me3 ChIP-seq dataset is part of the original Enformer dataset, for which the authors had already separated the test data from the training data by homology. Therefore, we used their splits without further processing. Training data were subsampled to 4164 sequences in order to keep the training sets balanced between all three species.

For the ATAC-seq data, we created only a train and validation split. This validation set was used for a small amount of hyperparameter tuning (see Section 2.3). Hyperparameters were transferred without tuning for the H3K4me3 ChIP-seq experiments, where we created a train and test split only.

### 2.3 Loss function, evaluation, and training details

All models were trained to predict track values from raw sequence training data using the Poisson negative log-likelihood as the loss function. Performance was then reported using the Pearson correlation coefficient on the validation or test set. The Pearson correlation coefficient was defined as $\text{Avg}((x-x_{\text{avg}})\cdot (y-y_{\text{avg}}))/\sqrt{\left(\text{Var}[x]\cdot \text{Var}[y]\right)}$, where *x* is the prediction and *y* is the target label value. We used the Poisson negative log-likelihood loss as the optimization target in training instead of directly using the Pearson correlation coefficient because the log-likelihood of a large dataset decomposes as a sum over the log-likelihoods of the individual examples and so is amenable to training using first-order stochastic optimization with small batch size, while the Pearson correlation coefficient is not.

To train the model, we used the AdamW optimizer (Loshchilov and Hutter 2017), which is the standard choice for deep neural network training. Except for the experiment in Fig. S5, available as supplementary data at *Bioinformatics* online, models were trained for 10 epochs. We tuned the learning rate and weight decay via grid search on the ATAC-seq validation data. For learning rate, we considered values in {1e−6, 3e−6, 1e−5, 3e−5, 1e−4, 3e−4}. For weight decay, we considered values in {1e−4, 3e−4, 1e−3}. We performed grid search over the ATAC-seq datasets and recorded the average validation Pearson correlation coefficient for each of the three species (cow, mouse, and pig) and for both fine-tuning and training from scratch. The most common optimal setting was a learning rate of 3e−5 and weight decay of 1e−4. In cases where this was not optimal, the correlation coefficient of the best-performing combination was <0.003 larger, which we deemed to be negligible. Therefore, we used this learning rate and weight decay setting for all other tasks with no further tuning, except for the experiment shown in Fig. 4; Fig. S4, available as supplementary data at *Bioinformatics* online. In that experiment, we further tuned the learning rate for fine-tuning using the DNase-seq myeloid progenitor task to 3e−7 and then used this learning rate for all other fine-tuning tasks in this experiment without further tuning. We used a linear learning rate warm-up from 0 to the peak in the first epoch and a linear learning rate decay from the peak to 0 in the later 9 epochs. We used a batch size of 1, as even a batch size of 2 did not fit into GPU memory.

Our results involve experiments in which models were pretrained on the same human genomic data used to train Enformer and then fine-tuned on one or a few genomic datasets from a nonhuman species, or in which models were trained from scratch only on datasets from a nonhuman species with the standard random initialization weights provided by Pytorch. Training was performed on a single Nvidia V100 GPU housed at Boston University’s Shared Compute Center. Models were implemented using Pytorch (Paszke *et al.* 2019).

### 2.4 Single-track and multi-track training

Our ATAC-seq datasets contain many tracks for each species. For these datasets, we trained in two modes: a “multitrack” mode in which the training objective is the average Poisson negative log-likelihood over all tracks and the reported metric is the average Pearson correlation coefficient over all tracks, and a “single-track” mode in which one individual track is selected. The training objective is the Poisson negative log-likelihood for just this selected track and the reported metric is the Pearson correlation coefficient for this same track. Our pretrained models, fine-tuned models, and trained from scratch models were all trained for 10 epochs.

### 2.5 Fine-tuning

To fine-tune a pretrained model on a nonhuman dataset, we loaded the pretrained checkpoint. Then, we deleted the last linear “head” layer that outputs predictions for each of the 5313 human tracks. We replaced this head layer with a new, randomly initialized, head layer that outputs *N* predictions where *N* is the number of tracks in the fine-tuning dataset. Finally, this model is trained on the nonhuman dataset.

In some experiments, we also froze the pretrained weights. This means that after loading the pretrained model from the checkpoint and replacing the head layer, we disabled gradient computation for all layers in the model except for the head layer. Then, during training, only the weights for the randomly initialized head layer were updated.

## 3 Results

### 3.1 Simplifying the Enformer model improves performance under computational constraints

We first investigated whether we could simplify the Enformer architecture such that it could be easily trained and fine-tuned on cheaper hardware without compromising the model’s performance. We specifically targeted the Nvidia V100 GPU, which is relatively accessible in the academic community. The published Enformer model consists of 7 convolutional residual blocks, 11 self-attention blocks, a pointwise convolutional layer, and finally 2 linear “head” layers, corresponding to human and mouse tracks, respectively (Avsec *et al.* 2021a). We considered several simplifications in which we removed individual layers or reduced the number of repeated blocks in Enformer (Fig. 1A).

Enformer was originally trained on both 5313 human datasets and 1643 mouse datasets (see Section 2). In order to test whether an Enformer-like model trained in one species can be applied to other species, we limited our training set to just the 5313 human datasets. We also reduced training time (10 epochs versus 150 epochs in the original study) and batch size (1 versus 64) to fit a smaller computational budget. Under these restrictions, the base Enformer model achieved a validation Pearson correlation value of 0.537.

First, we tested whether removing the final pointwise convolutional layer or the linear layer in each self-attention module would affect accuracy (Fig. 1B). Removing the final pointwise convolutional layer increased the Pearson correlation coefficient to 0.554 (“Enformer (no pointwise)”, purple in Fig. 1B), while removing the linear layer yielded a marginal increase to 0.540 (“Enformer (no linear)”, green in Fig. 1B). Removing both the final pointwise convolutional layer and linear layer in each self-attention module further improved the Pearson correlation coefficient to 0.565 (“Enformer (no linear, no pointwise)”, blue in Fig. 1B), while increasing the average iteration per second on a V100 GPU from 0.755 to 0.765. These changes resulted in a smaller, more efficient model that also achieved better prediction accuracy. This model architecture (“Enformer (no linear, no pointwise)”, blue in Fig. 1B) captured similar features when compared to the original Enformer model (Avsec *et al.* 2021a) (Fig. 1C) and was used for all subsequent experiments. We refer to this modified architecture as “simplified Enformer.”

Although training and test sets were carefully separated to avoid orthologous sequences as previously described (Avsec *et al.* 2021a), sequence homology between train and test sets may artificially boost model accuracy. To assess the model’s ability to generalize to sequences dissimilar to those in the training set, we grouped the test set into bins based on the proportion of each sequence with similarity to the training set (Fig. S2A, available as supplementary data at *Bioinformatics* online) and calculated individual Pearson correlation coefficients for these bins. As expected, the correlation coefficient increased with increasing sequence homology, but the magnitude of this effect was minor and was similar between the original Enformer model (Avsec *et al.* 2021a) and our simplified Enformer model (Fig. S2B, available as supplementary data at *Bioinformatics* online).

**Figure 2:**
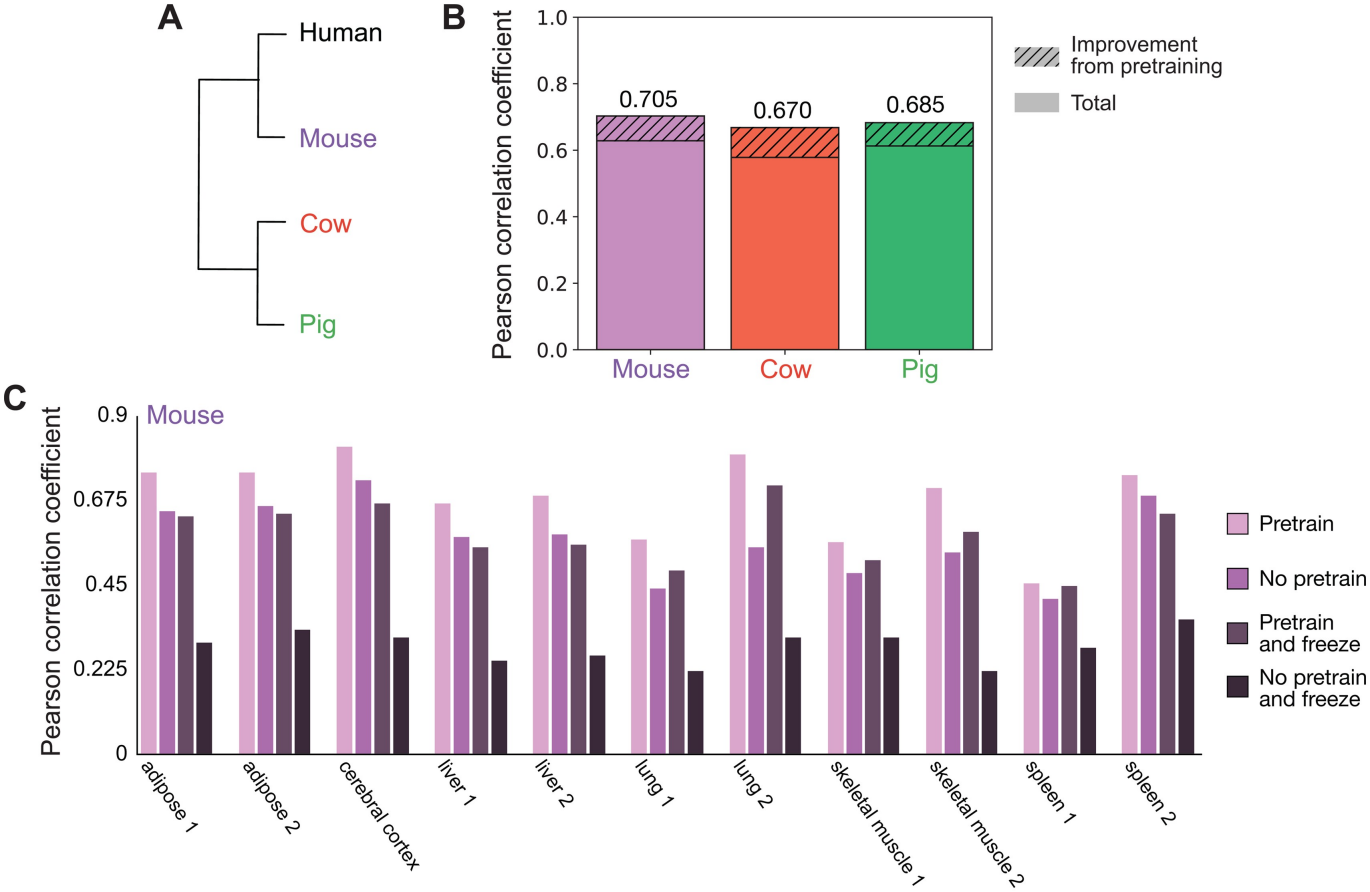
Pretraining improves performance on ATAC-seq datasets across multiple species. (A) Dendrogram of humans and species for which we examined previously generated ATAC-seq datasets (mouse, pig, cow). (B) Validation Pearson correlation coefficients for models trained on 11 mouse, 13 cow, or 13 pig ATAC-seq datasets (Halstead *et al*. 2020, Kern *et al*. 2021). The shaded regions are the difference between the Pearson correlation coefficient when using a model pretrained on human genomic datasets that is then fine-tuned on ATAC-seq datasets from mouse, cow, or pig and a model trained from scratch without pretraining. Total height indicates the Pearson correlation coefficient of the fine-tuned model. (C) Validation Pearson correlation coefficients for models trained on individual mouse ATAC-seq datasets. “Pretrain” is a model fine-tuned from a pretrained model. “No pretrain” is a model trained from scratch. “Pretrain and freeze” is a model fine-tuned from pretrained weights where only the last linear layer is updated in the fine-tuning process. “No pretrain and freeze” is a model trained from scratch where only the last linear layer is updated in the training process.

We also tested whether reducing the number of self-attention blocks in Enformer would affect model accuracy (“Enformer (5 layers, no linear, no pointwise”, yellow in Fig. 1). We found that 5 self-attention blocks, rather than the 11 used in Enformer, increased the iterations per second from 0.866 to 0.92, while maintaining a high Pearson correlation coefficient at 0.565. This suggests that there is a low marginal value for additional self-attention blocks at our computational budget. Although we used a model with 11 self-attention blocks in subsequent experiments because prior literature suggests that larger models may perform better for transfer learning (Hernandez *et al.* 2021), reducing the number of self-attention blocks would be a reasonable choice for highly resource-constrained researchers.

### 3.2 Pretraining on human data improves predictions on ATAC-seq datasets from nonhuman species

Using our simplified model architecture, we investigated whether fine-tuning a model pretrained on thousands of human datasets would improve accuracy in predicting ATAC-seq experiments from nonhuman species when compared to a model trained from scratch without pretraining (see Section 2). We generated predictions for 11 mouse, 13 cow, and 13 pig ATAC-seq datasets from diverse tissues including muscle, cerebellum, liver, and lung (Halstead *et al*. 2020, Kern *et al.* 2021) (Fig. 2).

Strikingly, pretraining improved the average Pearson correlation coefficients by 12.26% for mouse, 15.97% for cow, and 11.85% for pig (Fig. 2B). We then asked whether improvements from pretraining persist when fine-tuning on a *single* ATAC-seq dataset. We examined the same ATAC-seq datasets on a per-tissue basis (including replicates).

When training on a single ATAC-seq dataset, pretraining increased the validation Pearson correlation coefficient between experimental results and predictions from the model with an average improvement of 19.24%±10.83% (Fig. 2C; Fig. S3, available as supplementary data at *Bioinformatics* online, compare “Pretrain” to “No pretrain”). This suggests that pretraining on thousands of human datasets allows for high model accuracy even when fine-tuning on just a single dataset from a nonhuman species.

**Figure 3:**
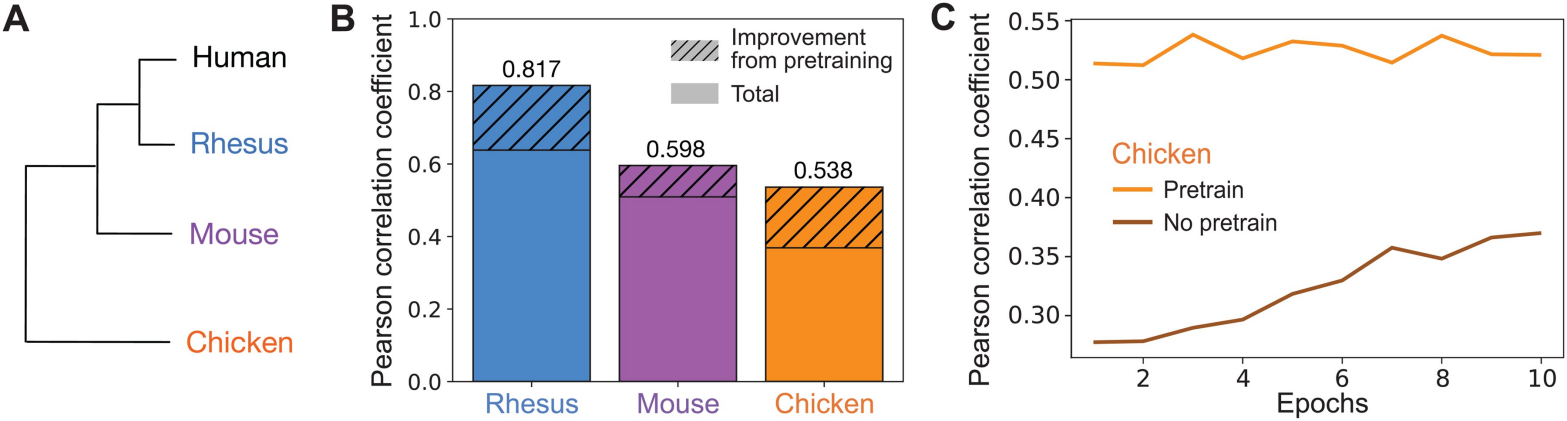
Pretraining improves performance on H3K4me3 ChIP-seq datasets across multiple species. (A) Dendrogram of humans and species where we examined previously generated H3K4me3 ChIP-seq datasets (rhesus, mouse, chicken). (B) Test Pearson correlation coefficients for models trained on a H3K4me3 ChIP-seq dataset from rhesus (Vermunt *et al*. 2016), mouse (Kelley *et al*. 2018), or chicken (Halstead *et al*. 2020). The shaded regions are the difference between the Pearson correlation coefficient when using a model pretrained on human genomic datasets that is then fine-tuned on a H3K4me3 ChIP-seq dataset in rhesus, mouse, or chicken (“cross-species pretrained model”) and a model without pretraining. Total indicates the Pearson correlation coefficient of the cross-species pretrained model. (C) Comparison of test Pearson correlation coefficients for the H3K4me3 ChIP-seq dataset from chicken across epochs with and without pretraining on human datasets.

Model accuracy varied widely between datasets both across and within species. Pretraining improved the correlation coefficient over training from scratch by 19.83%±11.69% for mice, 23.55%±12.51% for cows, and 14.43%±6.03% for pigs across single-dataset experiments. This is not correlated with evolutionary distance, as mice are more closely related to humans than cows. There was also dramatic variation across tissues and even within replicates of the same tissue within a species. For instance, the Pearson correlation coefficients for spleen_1, spleen_2, and cerebral cortex in mouse were 0.4511, 0.7388, and 0.8114, respectively. This inconsistency in prediction accuracy not only for different species or for different tissues within the same species but also for replicates of the same tissue in the same species suggests that differences in model accuracy are due to technical features of the experimental datasets and not due to biological differences between tissue types or species.

Next, we considered freezing the pretrained layers (i.e. not updating the parameters and only updating the last linear layer) on a single ATAC-seq dataset. This is a standard strategy for efficient fine-tuning (Yosinski *et al.* 2014), and if it could achieve comparable effects to “full” fine-tuning, then it would decrease the computational resources on a V100 GPU: with freezing, training took 12 h with 2.49 GB peak CUDA memory usage, compared to 28 h and 9.50 GB without freezing. As a control, we also performed no pretraining (i.e. random initialization) and updated only the last linear layer on a single ATAC-seq dataset, which performed poorly as expected (“No pretrain and freeze” in Fig. 2C; Fig. S3, available as supplementary data at *Bioinformatics* online). Freezing the pretrained layers and only updating the last linear layer on a single ATAC-seq dataset (“Pretrain and freeze” in Fig. 2C; Fig. S3, available as supplementary data at *Bioinformatics* online) dramatically out-performed the control group and performed comparably to training from scratch on a single ATAC-seq dataset. However, it did not perform as well as using a pretrained model and then fine-tuning the entire model (“Pretrain” in Fig. 2C; Fig. S3, available as supplementary data at *Bioinformatics* online). Thus, although updating only the last linear layer may be suitable in truly resource-limited environments, pretraining followed by fine-tuning the entire model produces the highest model accuracy.

### 3.3 Pretraining improves predictions and time on H3K4me3 ChIP-seq datasets across species

We then asked whether the improvement in model accuracy from pretraining is generalizable to other types of genomic datasets and to nonmammalian species. We examined ChIP-seq datasets for H3K4me3, a histone mark commonly found at promoters and enhancers (Shlyueva *et al.* 2014), that were generated from rhesus macaque, mouse, and chicken cerebellum (Vermunt *et al*. 2016, Kelley *et al.* 2018, Halstead *et al*. 2020) (Fig. 3A). To ensure that our findings in the ATAC-seq experiments were not a result of over-fitting on the validation set, we directly applied the hyperparameters used in the ATAC-seq experiments to experiments with H3K4me3 data without additional hyperparameter tuning.

Consistent with our ATAC-seq results, pretraining significantly improved model accuracy by 28.03%, 17.49%, and 46.09% for rhesus, mouse, and chicken, respectively (Fig. 3B). Like the ATAC-seq datasets, improvements in model accuracy from pretraining on human datasets were not correlated with evolutionary distance from humans, with chickens having the largest improvement in test accuracy from pretraining. This suggests that pretraining even in a distantly related species can still dramatically improve model performance upon fine-tuning in a species of interest.

Notably, pretraining also significantly reduces computation time. For both ATAC-seq and H3K4me3 ChIP-seq datasets, we found that even after a single epoch of fine-tuning, the correlation coefficient was already higher than after 10 epochs of training from scratch (see Fig. 3C for a representative plot). Thus, pretraining allows us to exceed the performance of a nonpretrained model using a >10× smaller computational budget, and significantly improves model performance across diverse genomic datasets, tissues, and species.

### 3.4 Training on excessive data decreases model performance

**Figure 4:**
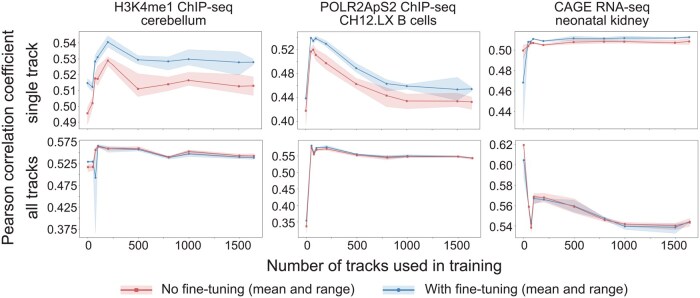
Training on an excessive number of tracks hinders model performance. We trained our simplified Enformer model on a track of interest and 1, 50, 75, 100, 200, 500, 800, 1000, 1500, and 1642 randomly selected additional tracks for 10 epochs. Test Pearson correlation coefficients for just the track of interest (red line—top) or across all tracks (red line—bottom) are plotted. We then fine-tuned the models for another 10 epochs on just the track of interest and reported the fine-tuned test Pearson correlation coefficients (blue lines). Results are shown here for three tracks of interest. Results for six additional tracks of interest are shown in Fig. S4, available as supplementary data at *Bioinformatics* online.

In machine learning, training on multiple tasks can improve the model’s generalizability. However, excessive learning on diverse training tasks can impair performance on the task of interest by distributing the model’s learning ability across multiple tasks, leading to a tradeoff between generalization and specialization. To determine whether there are tradeoffs between generalization and specialization when training on diverse genomic datasets, we trained our simplified Enformer model on an increasing number of tracks and examined its average performance across all tracks and on a specific track of interest. We arbitrarily chose nine tracks of interest, of which three representative tracks are shown in Fig. 4 and the remainder in Fig. S4, available as supplementary data at *Bioinformatics* online.

We trained the Enformer model on the track of interest and 1, 50, 75, 100, 200, 500, 800, 1000, 1500, and 1642 additional tracks that were randomly selected for each track of interest. We performed three replicates for each track count, initializing the model randomly in each replicate. We found that the average Pearson correlation coefficient across all tracks is typically highest when using 100–200 tracks for training, but occasionally may peak at a single additional track, likely due to the one added track being a particularly easy prediction task (as seen for CAGE RNA-seq in Fig. 4). On our track of interest, we observed the highest correlation at 100–200 tracks for training. When the number of tracks used during training increased beyond 200, the correlation coefficient either began decreasing or at best saturated, indicating that excessive data are not beneficial and may even hinder the model’s ability to learn specific tasks.

To investigate whether fine-tuning can improve a model’s specialization after training on an excessive number of tasks, we fine-tuned the models for an additional 10 epochs on the single track of interest. As expected, fine-tuning improved the Pearson correlation coefficient for the track of interest (top plots in Fig. 4; Fig. S4A, available as supplementary data at *Bioinformatics* online). Strikingly, however, fine-tuning could not overcome the negative impact of training on an excessive number of tasks, as performance decreased when the number of tracks used in training was greater than 200 with or without fine-tuning. Of note, fine-tuning had little effect on performance when examining the average Pearson correlation coefficient across all tracks (bottom plots in Fig. 4; Fig. S4B, available as supplementary data at *Bioinformatics* online). These findings suggest that while fine-tuning can improve performance, carefully calibrating the number of training tasks is critical for achieving peak performance.

## 4 Discussion

This work focuses on improving the efficiency and applicability of large deep learning models for predicting genomic datasets. We make contributions along three axes: modification of model architecture for cheaper training, using pretraining to train more effectively on single datasets, and showing that more training data does not necessarily result in more accurate models.

First, we find that deleting a linear layer from each self-attention block and a final pointwise convolutional layer from the original Enformer model improved model accuracy. We chose to ablate these layers because they conceptually reflect processing on a per-locus level that might be plausibly replicated in the attention layers. It is possible that these particular modules were only marginally useful for representing a good prediction value while significantly increasing parameter count. Since classical statistics suggests that increased parameter count can increase training difficulty (Wainwright 2019), this may explain the advantage of removing these layers. It remains to be determined whether these modifications would also improve the Enformer model when it is trained in the computationally intensive setting used by the original authors (Avsec *et al.* 2021a).

Although these modifications resulted in improvements over the baseline Enformer model at our smaller computational budget, this simplified model did not reach the 0.625 Pearson correlation coefficient reported by the Enformer authors (Avsec *et al.* 2021a) when trained on both human and mouse datasets. This gap is likely due to our significantly constrained compute budget (10 versus 150 epochs), as well as not using mouse data during training. Accordingly, when we trained our simplified Enformer model for more epochs, the Pearson correlation coefficient improved from 0.561 at 10 epochs to 0.583 at 50 epochs (Fig. S5, available as supplementary data at *Bioinformatics* online), suggesting that additional epochs and training data may further improve performance. Nevertheless, we find that using the initial weights from our simplified Enformer model pretrained on human datasets to then fine-tune on nonhuman datasets significantly improved performance over training from scratch and allowed us to reach similar or higher Pearson correlation coefficients (as high as 0.8 in many cases). This demonstrates that state-of-the-art predictions can be achieved even on a limited computational budget by tailoring the model architecture and using pretraining.

We posit that additional modifications to the model architecture may further improve performance. For instance, parameter-efficient fine-tuning strategies such as low-rank adaptation (Hu et al. 2021) are now popular and effective techniques for fine-tuning large language models in natural language processing, and could be of potential use in genomics as well. In addition, recent alternatives to attention-based architectures based on state-space models have proved effective on unsupervised DNA modeling and enhancer prediction tasks (Nguyen *et al.* 2023), suggesting that more dramatic architectural modifications may also prove effective.

Further, we show that as the number of tracks used during training increases, the correlation coefficient starts to decrease, indicating that excessive data can hinder the model’s learning ability and reduce overall performance. A similar phenomenon named the “loss of plasticity,” where learning from an excessive number of previous tasks can affect the agent’s ability to adapt to novel tasks, is also observed in reinforcement learning (Abbas *et al.* 2023). Our findings from fine-tuning on a single track of interest suggest that while fine-tuning helps to improve the model’s performance after training on excessive data, it does not fully mitigate the tradeoff between generalization and specialization (i.e. the model’s ability to perform well on a specific task). Thus, training on all available data followed by fine-tuning on a specific task may not be the optimal strategy for balancing both specialization and generalization.

## Supplementary Material

btag139_Supplementary_Data

## Data Availability

No new data were generated in support of this research. The data analyzed in this article and how to access them are referenced in the article or its online supplementary material.
