# Supplementary material for: Pretraining improves prediction of genomic datasets across species
Source: Bioinformatics. 2026 Mar 23;42(4):btag139. doi: 10.1093/bioinformatics/btag139 (PMC13070385; doi:10.1093/bioinformatics/btag139)
Supplement: btag139_Supplementary_Data [file btag139_supplementary_data.pdf]

Supplement: Pretraining Improves Prediction of Genomic Datasets Across Species

Fangrui Huang[1,2†], Yitong Wang[1†], Ashok Cutkosky[3*], Janet H.T. Song[4*]

[1] Department of Computer Science, Boston University
[2] Department of Computer Science, Stanford University
[3] Department of Electrical and Computer Engineering, Boston University
[4] Department of Human Evolutionary Biology, Harvard University
[†] Equal contribution
[*] Corresponding authors: cutkosky@bu.edu, janetsong@fas.harvard.edu

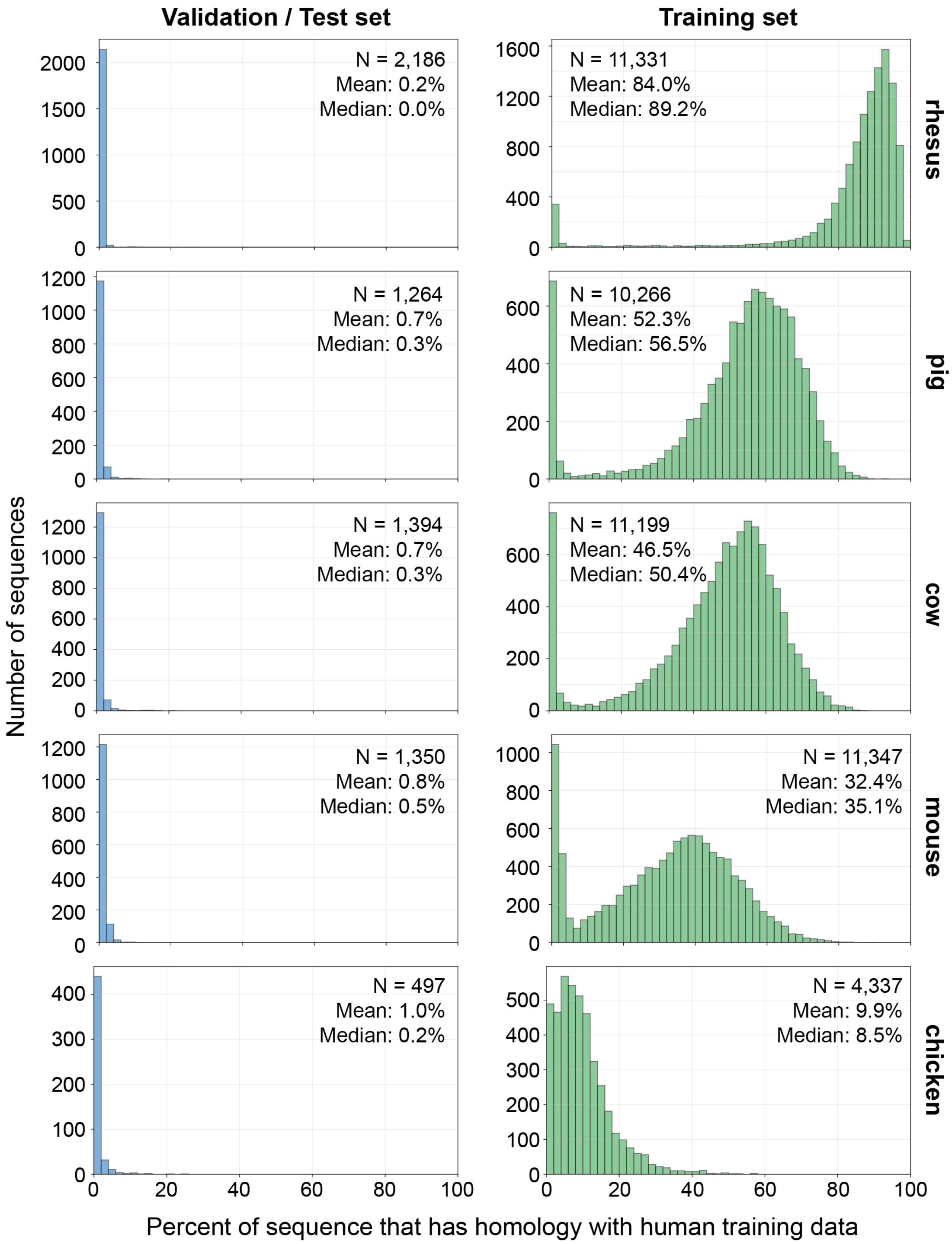

Figure S1: Homology between human training sequences and sequences in validation/test and training sets for non-human species. Sequences partitioned to validation/test and training sets for each non-human species (rhesus macaque, pig, cow, mouse, and chicken) were aligned to the human genome using pslMap (Zhu et al., 2007). The percentage of base pairs in each sequence that aligned to any human training sequence is plotted. Validation/test sequences have very low homology to the human training sequences, while training sequences display roughly increasing homology as the evolutionary distance between the target species and humans decreases. These training sets were then sub-sampled so that the same number of sequences were used across species for ATAC-seq and ChIP-seq experiments, respectively (Methods). Note that validation/test sets were used as validation sets for ATAC-seq and as test sets for ChIP-seq, as described in Methods.

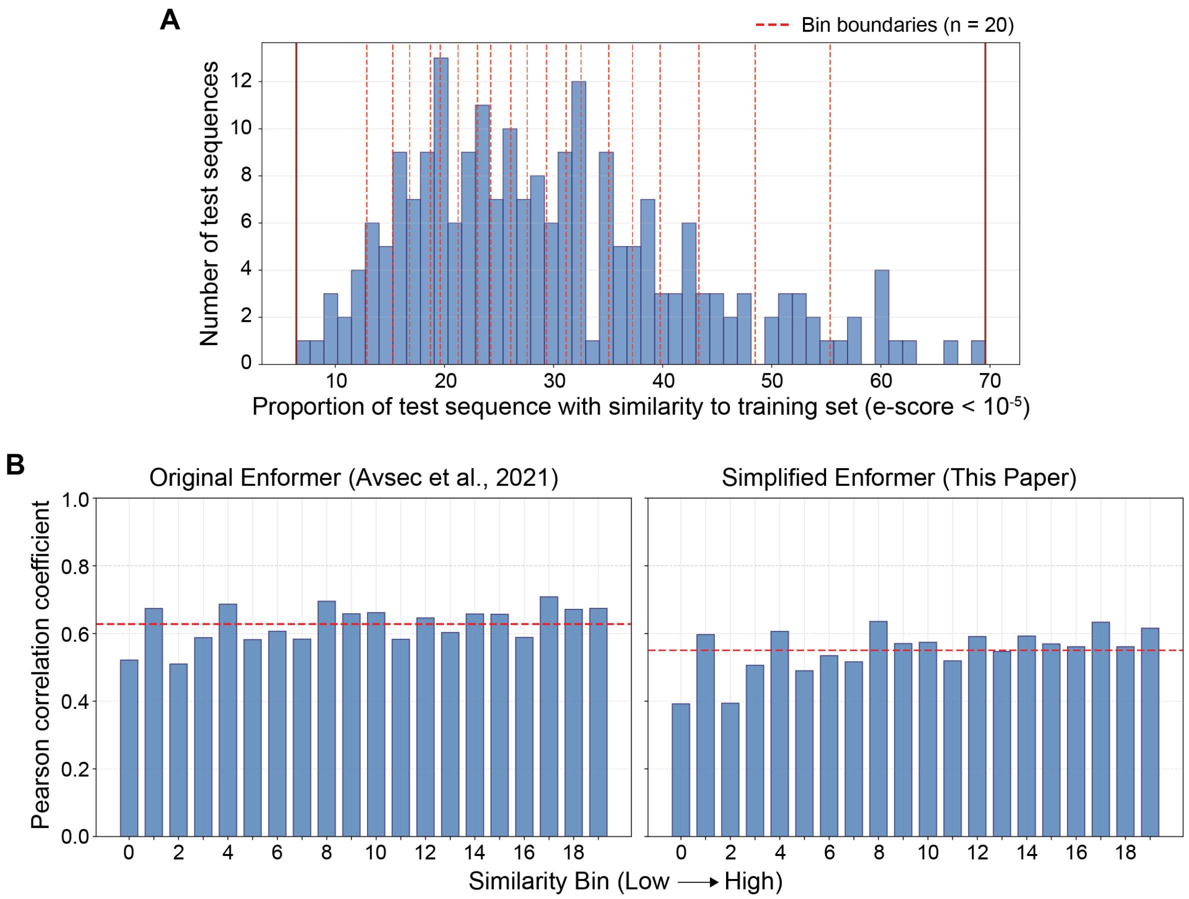

Figure S2: Homology stratification of human test set. (A) We calculated the proportion of each test sequence with e-score $< 10^{-5}$, indicating sequence similarity, using BLAST against sequences in the training set. Test sequences were grouped into 20 quantile-based bins, each containing 5% of the test sequences. (B) The Pearson correlation coefficient increased with increasing sequence homology, but the magnitude of this effect was minor and was similar between the original Enformer model (Avsec et al., 2021) and our simplified Enformer model ($\Delta r = 0.22$ and 0.15 between lowest and highest homology bins for the original Enformer model (Avsec et al., 2021) and our simplified Enformer model, respectively).

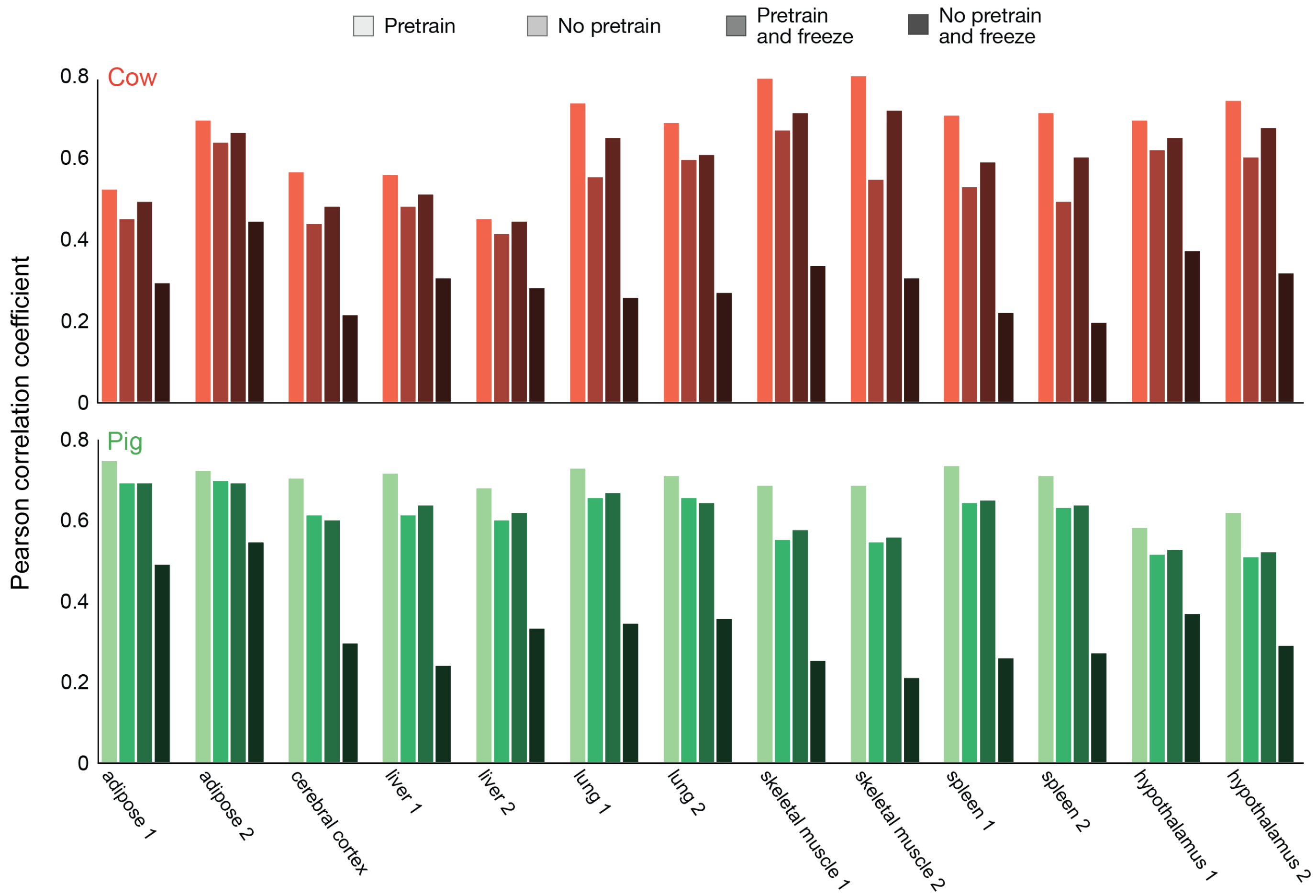

Figure S3: Pretraining improves performance on ATAC-seq datasets across multiple species. Validation Pearson correlation coefficients for models trained on individual ATAC-seq datasets from cow and pig. "Pretrain" is a model fine-tuned from a pretrained model. "No pretrain" is a model trained from scratch. "Pretrain and freeze" is a model fine-tuned from pretrained weights where only the last linear layer is updated in the fine-tuning process. "No pretrain and freeze" is a model trained from scratch where only the last linear layer is updated in the training process.

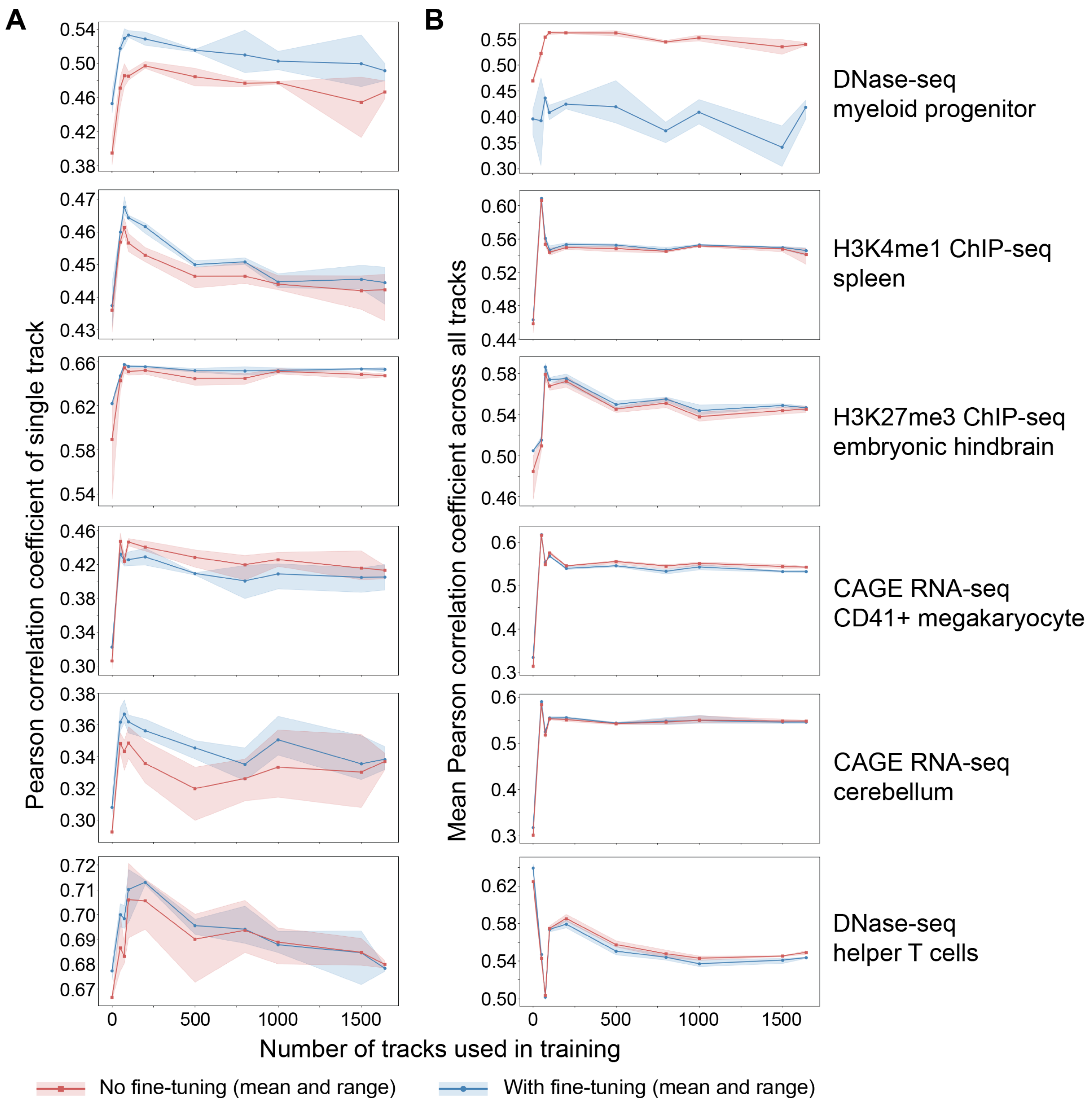

Figure S4: Training on an excessive number of tracks hinders model performance. We trained our simplified Enformer model on a track of interest and 1, 50, 75, 100, 200, 500, 800, 1000, 1500, and 1642 additional tracks for 10 epochs. The added tracks were randomly selected for each track of interest, and three replicates were performed for each track of interest. The Pearson correlation coefficient for just the track of interest (red line - left) or across all tracks (red line - right) is plotted. We then fine-tuned the models for another 10 epochs on just the track of interest and reported the fine-tuned Pearson correlation coefficients (blue lines). We tuned the learning rate on the "DNase-seq myeloid progenitor" task, and so reported values serve as validation scores. The other tasks used the hyperparameters from the "DNase-seq myeloid progenitor" task, and so are test scores.

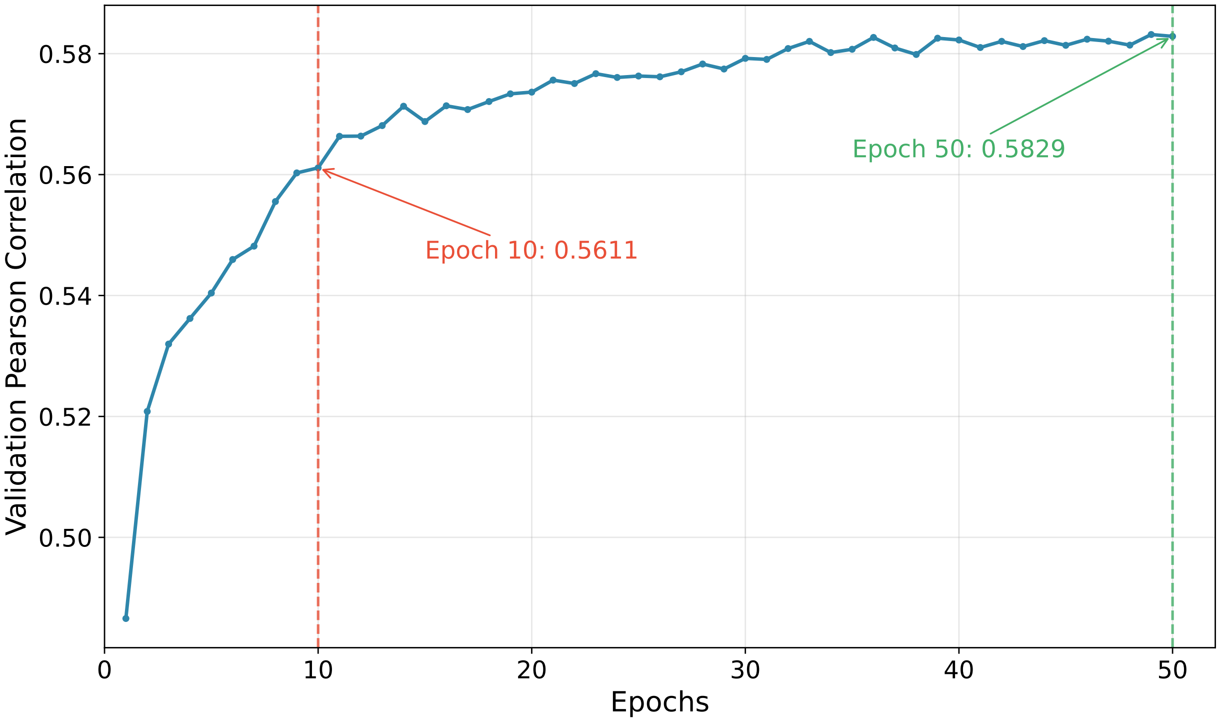

Figure S5: Performance of simplified Enformer model when trained for 50 epochs. Validation Pearson correlation coefficient of the simplified Enformer model (“Enformer (no linear, no pointwise)”, blue in Figure 1B) trained over 50 epochs.

References

Žiga Avsec, Vikram Agarwal, Daniel Visentin, Joseph R Ledsam, Agnieszka Grabska-Barwinska, Kyle R Taylor, Yannis Assael, John Jumper, Pushmeet Kohli, and David R Kelley. Effective gene expression prediction from sequence by integrating long-range interactions. *Nature Methods*, 18(10):1196–1203, 2021.

Jingchun Zhu, J Zachary Sanborn, Mark Diekhans, Craig B Lowe, Tom H Pringle, and David Haussler. Comparative genomics search for losses of long-established genes on the human lineage. *PLoS Comput Biol*, 3(12):e247, Dec 2007.
